# Digital Surgical Management of Traumatic Occlusal Disorder: A Case Report

**DOI:** 10.1155/crid/9103011

**Published:** 2026-10-21

**Authors:** Yuwei Liang, Qian Jiang, Junhui Ma, Yewei Xin, Feng Qiu

**Affiliations:** ^1^ Guilin Medical University Guilin Guangxi China glmc.edu.cn; ^2^ Affiliated Stomatological Hospital of Guilin Medical University Guilin Guangxi China; ^3^ The First Affiliated Hospital of Zhengzhou University Zhengzhou Henan China zzu.edu.cn

**Keywords:** case report, digital surgery, mandibular fracture, occlusal disorder, virtual surgical planning

## Abstract

**Background:**

Traumatic occlusal disorders secondary to complex mandibular fractures, particularly those involving the condylar region, present a dual challenge: achieving anatomical reduction while restoring physiological function. Conventional surgical approaches are constrained by limited access, soft tissue swelling, and the inherent difficulty of occlusal reconstruction.

**Case Presentation:**

A 33‐year‐old male presented with an anterior open bite 3 days after maxillofacial trauma. Clinical examination revealed limited mouth opening with preserved facial symmetry. Computed tomography confirmed a sagittal fracture of the right mandibular premolar region and a displaced left condylar neck fracture. A comprehensive digital surgical workflow was deployed, integrating virtual surgical planning, 3D‐printed occlusal splint fabrication, and intraoperative guided fracture fixation. Postoperative outcomes demonstrated stable occlusal contact, anatomical condylar reduction, and satisfactory functional recovery.

**Conclusion:**

The integration of digital surgical techniques enables precise, function‐oriented reconstruction in complex mandibular fractures. This approach aligns with the concept of “physiological reduction” and provides a reproducible strategy for managing traumatic occlusal disorders.

## 1. Introduction

Mandibular fractures are among the most common injuries encountered in maxillofacial trauma practice, with condylar fractures accounting for a substantial proportion of these cases. The reported prevalence of condylar process fractures varies widely across the literature, ranging from 16.5% to 56%, with most estimates falling between 25% and 35% [1, 2]. Kozakiewicz et al. recently reported that among 386 patients with mandibular fractures, condylar process fractures constituted 45% of all fractures, with basal fractures being the most common subtype (54%) and mandibular head fractures comprising 34% [2].

The optimal management of condylar fractures remains contentious. Historically, closed reduction with intermaxillary fixation or functional therapy was the mainstay, particularly for intracapsular fractures. However, accumulating evidence suggests that open reduction and internal fixation (ORIF) may provide superior outcomes in selected fracture patterns, especially subcondylar and high‐neck fractures. A meta‐analysis comparing closed treatment with ORIF for condylar head fractures found that ORIF was associated with significantly lower odds of postoperative occlusal discrepancy (OR 0.14; 95% CI 0.03–0.79) [3]. Another meta‐analysis reported better temporomandibular joint (TMJ) function and less malocclusion with ORIF [4]. Nevertheless, the overall certainty of the evidence remains low, and both treatment strategies continue to be regarded as acceptable.

The mandibular condyle resides in a deep, confined anatomical space, intimately related to the facial nerve and maxillary vessels, rendering surgical precision indispensable [5]. Suboptimal outcomes can result in persistent pain, malocclusion, restricted mandibular movement, ankylosis, and chronic TMJ dysfunction, all of which profoundly impair patients′ quality of life [6]. The central challenge in managing these injuries is achieving a harmonious balance between anatomical reconstruction and functional restoration—a concept increasingly referred to as “physiological reduction” [7].

Digital surgical technologies, including virtual surgical planning (VSP) and three‐dimensional (3D) printing, have transformed maxillofacial trauma care. VSP enables preoperative simulation of fracture reduction, allowing surgeons to anticipate complications and establish precise functional endpoints [8]. 3D printing facilitates the fabrication of patient‐specific occlusal splints and precontoured plates, enhancing intraoperative accuracy while reducing surgical complexity [9, 10]. A systematic review confirmed that 3D printing significantly improves precision, shortens operative time, and enhances aesthetic outcomes in mandibular fracture management [11]. Despite these advances, the application of these technologies in acute trauma settings remains challenging, particularly with respect to rapid turnaround and seamless workflow integration [12].

This case report describes the use of a comprehensive digital surgical workflow—from conventional impression‐taking to 3D‐printed occlusal splint fabrication—for a complex mandibular fracture with traumatic occlusal disorder and demonstrates its feasibility and advantages in achieving physiological reduction.

## 2. Case Presentation

This case report was prepared in accordance with the CARE checklist (CARE_Checklist_Supplementary (Available here)).

### 2.1. Patient Information

A 33‐year‐old male presented to the Affiliated Stomatological Hospital of Guilin Medical University with an inability to occlude normally 3 days after sustaining blunt facial trauma. He had no significant past medical history. There was no relevant family or psychosocial history.

### 2.2. Clinical Examination

Examination revealed an anterior open bite, inability to chew, and limited mouth opening. Facial symmetry was preserved (Figure 1), and no facial nerve dysfunction was noted.

**Figure 1:**
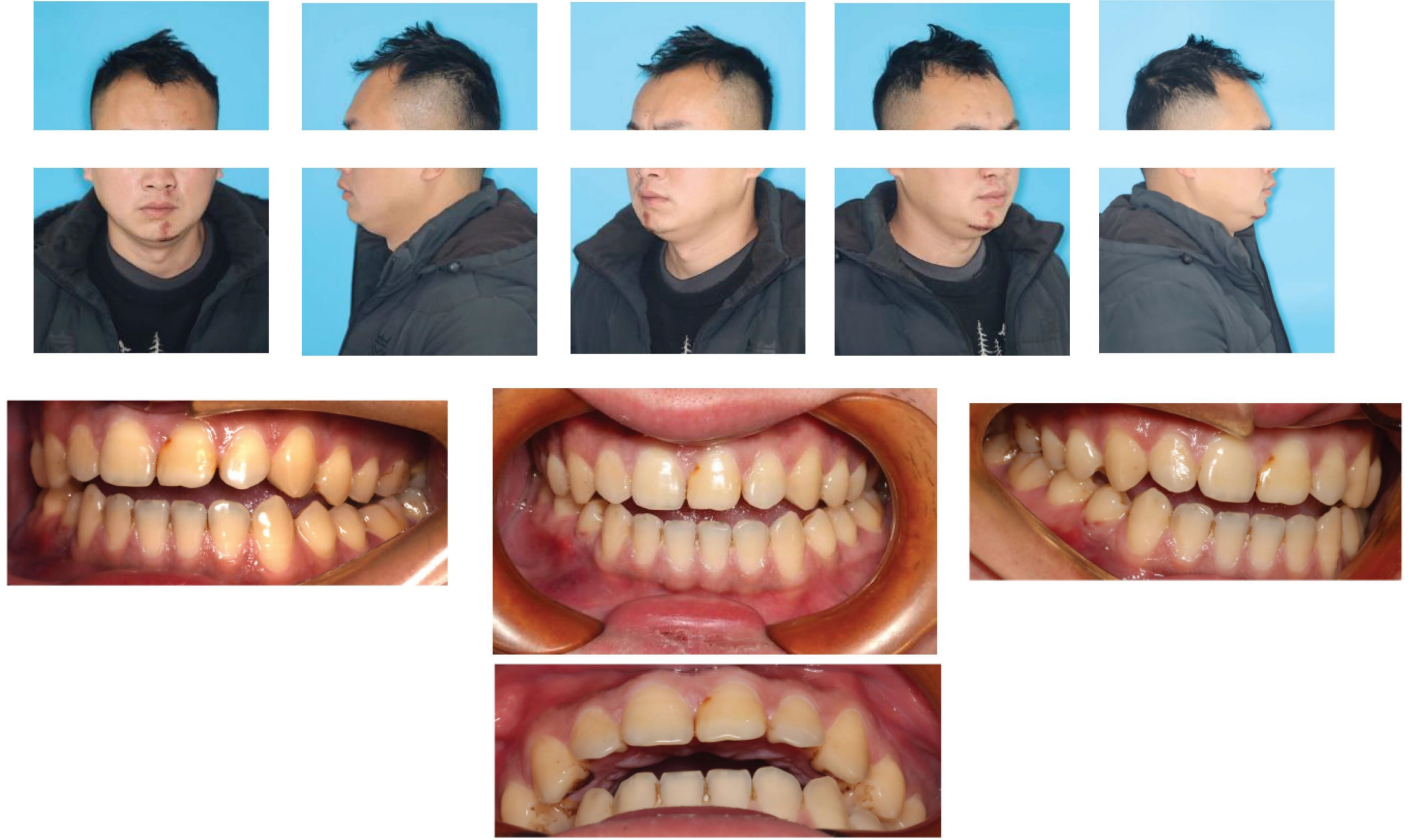
Preoperative clinical photographs showing anterior open bite and facial symmetry.

### 2.3. Imaging Findings

Computed tomography (CT) showed a sagittal fracture of the right mandibular premolar region and a displaced left condylar neck fracture (Figure 2). No other fractures were identified.

**Figure 2:**
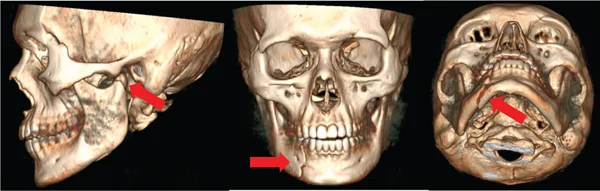
Preoperative CT images demonstrating sagittal fracture in the right mandibular premolar region and displaced left condylar neck fracture.

### 2.4. Digital Surgical Workflow

A stepwise digital surgical workflow was implemented:

Step 1—Model preparation: Dental impressions were obtained and plaster models were fabricated. Fracture lines were simulated, and the model was rearticulated to achieve an ideal occlusal relationship (Figure 3A).

**Figure 3:**
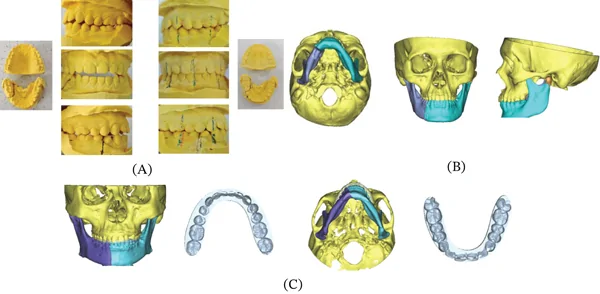
Digital surgical planning workflow: (A) plaster model preparation and occlusal reconstruction, (B) virtual fracture reduction simulation, and (C) occlusal splint design.

Step 2—Digital acquisition: The plaster model was digitized using a dental scanner to generate a high‐resolution 3D digital model (Figure 3B).

Step 3—3D reconstruction: CT data were fused with the digital dental model to create a detailed craniofacial reconstruction (Figure 3B).

Step 4—Virtual planning: Fracture reduction was simulated using MIMICS software to define optimal bone segment positioning, occlusal relationships, and condylar position (Figure 3C).

Step 5—Occlusal splint fabrication: A patient‐specific occlusal splint was designed and fabricated using 3D printing technology. This device functioned as an occlusal positioning splint rather than a bone‐borne surgical guide; it was used to establish the ideal maxillomandibular relationship and to indirectly guide the condylar segment into the glenoid fossa (Figure 3C).

### 2.5. Surgical Procedure

The procedure was performed under general anesthesia as follows:

1. Eight traction screws were placed intraorally.
2. The right mandibular body fracture was exposed through a right intraoral mandibular vestibular incision (Figure 4A).
3. The left condylar neck fracture was exposed through a left preauricular incision, with careful protection of the facial nerve branches (Figure 4C).
4. The 3D‐printed occlusal splint was secured to the maxillary dentition (Figure 4B).
5. The mandible was repositioned to engage the splint accurately, establishing the preoperatively planned occlusal relationship.
6. Intermaxillary fixation was applied.
7. The right mandibular body fracture was reduced and fixed first using titanium miniplates and screws.
8. Under the guidance of the established occlusion, the left condylar fragment was anatomically reduced into the glenoid fossa and fixed with a titanium miniplate (Figure 4D).
9. After removal of the splint, occlusal verification was performed immediately (Figure 4E–F).
10. Condylar seating within the glenoid fossa was confirmed intraoperatively.
11. Postoperative intermaxillary elastic traction was applied to maintain the occlusal relationship and TMJ vertical dimension during early healing.

**Figure 4:**
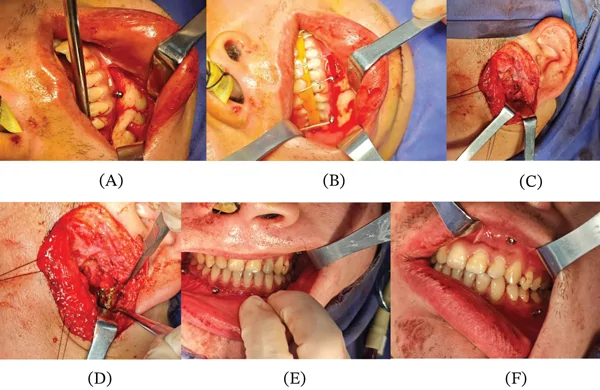
Intraoperative photographs. (A) Exposure of the right mandibular body fracture via a right intraoral vestibular incision. (B) The 3D‐printed occlusal splint was secured to the maxillary dentition for intermaxillary fixation. (C) Exposure of the left condylar neck fracture via a left preauricular incision (note the left ear). (D) Anatomical reduction and internal fixation of the left condylar fracture. (E–F) Immediate occlusal verification after removal of the splint, demonstrating stable and reproducible occlusal contacts.

## 3. Results

### 3.1. Intraoperative Outcomes

Stable and reproducible occlusion was achieved, consistent with the preoperative virtual plan. The condyle was anatomically reduced without evidence of displacement.

### 3.2. Postoperative Imaging

Postoperative CT confirmed anatomical reduction of both fractures, with symmetric distribution of TMJ loading (Figure 5). No malunion or nonunion was observed.

**Figure 5:**
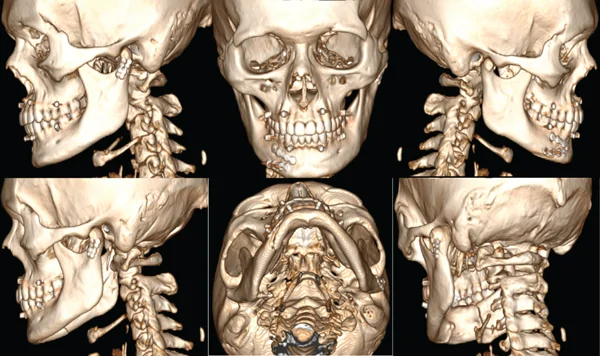
Postoperative CT images confirming anatomical reduction of right mandibular fracture and left condylar fracture.

### 3.3. Functional Recovery

Intermaxillary elastic traction was applied during the early healing phase. At follow‐up, the patient demonstrated restored jaw function without occlusal discomfort, stable occlusion, satisfactory facial symmetry, and improved mouth opening (Figure 6). The patient reported satisfaction with the facial appearance and occlusal function at follow‐up.

**Figure 6:**
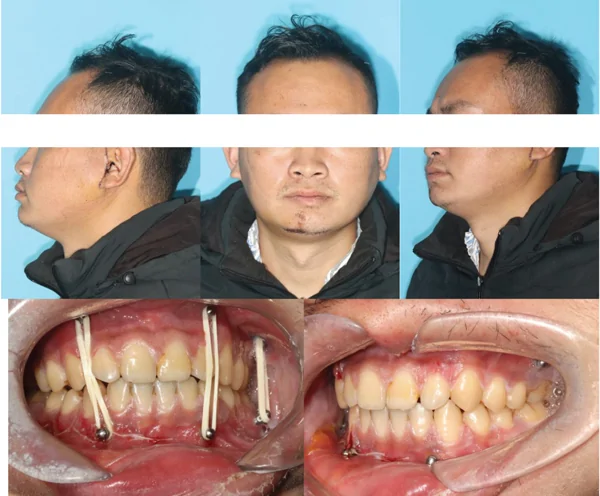
Postoperative clinical photographs demonstrating restored occlusal contact and facial symmetry.

## 4. Discussion

This case demonstrates that the integration of VSP, 3D printing, and intraoperative guided fixation can achieve precise, function‐oriented reconstruction in a complex two‐site mandibular fracture—a result consistent with the concept of “physiological reduction” [7]. Beyond demonstrating technical feasibility, this report provides several practical insights that may assist surgeons in adopting similar digital workflows for challenging mandibular fractures.

### 4.1. Clinical Challenges and Digital Solutions

The fracture pattern in this case—a sagittal body fracture combined with a displaced contralateral condylar neck fracture—presents unique difficulties. The sagittal component disrupts the continuity of the mandibular basal arch, whereas the condylar neck fracture destabilizes the vertical ramus. In a conventional surgical setting, the operator is required to reduce two independent fracture sites without a stable reference point for either, often relying on repeated trial‐and‐error manipulation and intermittent occlusal checks.

The digital workflow addressed this challenge by establishing a unified functional endpoint—the preoperatively defined occlusal relationship—before any surgical intervention. The 3D‐printed occlusal splint served as a physical transfer of this virtual plan, enabling both fracture fragments to be reduced toward the same occlusal target. This approach minimized intraoperative uncertainty and reduced the need for repeated manipulation of bone fragments, which may contribute to better preservation of fragment vascularity and surrounding soft tissue integrity.

### 4.2. Practical Considerations for Clinical Implementation

From our experience with this case, several technical considerations may be relevant for surgeons planning to adopt a similar digital pathway.

First, the accuracy of the printed occlusal splint is directly dependent on the quality of the initial plaster model articulation. We observed that dedicating additional time to this preparatory step—ensuring that the simulated occlusal relationship faithfully represented the patient′s pre‐injury dental architecture—was fundamental to the success of all subsequent phases.

Second, the mechanical design of the occlusal splint must be sufficiently robust to withstand the forces applied during intermaxillary fixation. In our case, incorporating a palatal extension into the splint design significantly enhanced anchorage stability.

Third, immediate occlusal verification after splint removal proved to be an indispensable intraoperative quality‐control measure. In our patient, the occlusion remained stable after splint removal, thereby confirming the accuracy of the entire digital chain. Had instability been detected, the surgeon would have retained the opportunity to revise the reduction before final fixation—a distinct advantage over workflows that lack such a verification step.

### 4.3. Context Within the Literature

The management of condylar fractures continues to generate debate. In the present case, the presence of a displaced condylar neck fracture with an associated mandibular body fracture strongly favored surgical intervention. A meta‐analysis by Rashid et al. reported that ORIF for condylar head fractures was associated with a lower risk of postoperative occlusal discrepancy (OR 0.14), although the gain in maximal mouth opening was modest (mean difference 2.72 mm) [3]. The same study also found an 11% rate of TMJ‐related morbidity following closed treatment, which further supports the surgical approach in appropriately selected patients.

The anatomical constraints of the condylar region are well recognized [5, 13]. Standard preauricular approaches carry a 3% risk of facial nerve weakness, which increases to 23% with modified approaches [14]. In this context, the digital workflow offered a practical advantage by enabling detailed preoperative spatial planning without additional radiation exposure and by allowing precise execution of the planned occlusion without repetitive intraoperative manipulation. The left preauricular incision required careful dissection to protect the temporal branches of the facial nerve, whereas the right intraoral approach avoided external scarring and facial nerve injury. The vertical dimension of the TMJ was maintained by the occlusal splint, which prevented collapse of the condylar segment; postoperative elastic traction further supported the condyle during healing.

### 4.4. Limitations

Several limitations of this report should be acknowledged. As a single case study, the findings are not generalizable without validation in larger case series or controlled studies. Longer‐term follow‐up is required to fully evaluate TMJ function and masticatory efficiency. Additionally, the cost and time associated with VSP and splint fabrication may limit the widespread adoption of this approach, particularly in resource‐constrained or acute trauma settings [12]. Nevertheless, as office‐based digital workflows become increasingly accessible [6], these barriers are likely to diminish over time. Ultimately, digital tools should be viewed as complements to, rather than substitutes for, sound surgical judgment and thorough anatomical knowledge.

## 5. Conclusions

VSP and 3D‐printed occlusal splints offer accuracy and predictability that enable a shift from empirical fixation toward personalized functional reconstruction. This case confirms that a comprehensive digital workflow—encompassing virtual planning, splint fabrication, and guided fixation—can achieve stable occlusion, anatomical condylar reduction, and satisfactory functional recovery in complex mandibular fractures with traumatic occlusal disorder (Figures 5 and 6). This approach aligns with the concept of “physiological reduction” and provides a reproducible model for management. Future research should focus on long‐term outcomes, improved accessibility, and integration of these technologies into routine trauma protocols.

## Author Contributions

Yuwei Liang: conceptualization, investigation, writing – original draft. Qian Jiang: data curation, visualization. Junhui Ma: methodology, validation. Yewei Xin: resources, software. Feng Qiu: supervision, project administration, writing – review and editing.

## Funding

This study was supported by the Western China Stomatological Clinical Research Fund of the Chinese Stomatological Association, No. CSA‐W2024‐12.

## Disclosure

All authors have read and agreed to the published version of the manuscript.

## Ethics Statement

This study was conducted in accordance with the Declaration of Helsinki and approved by the Ethics Committee of the Affiliated Stomatological Hospital of Guilin Medical University (Approval No. KQ‐LL2024001). Written informed consent was obtained from the patient for the publication of this case report and any accompanying images.

## Consent

Written informed consent was obtained from the patient for the publication of this case report and any accompanying images. The patient was informed that his anonymity would be preserved, and he was given the opportunity to withdraw his consent at any time prior to publication.

## Conflicts of Interest

The authors declare no conflicts of interest.

## Supporting information


**Supporting Information** Additional supporting information can be found online in the Supporting Information section. The CARE checklist was used to ensure transparent and complete reporting of this case. The completed checklist is provided as Supporting Information (CARE_Checklist_Supplementary).

## Data Availability

Data sharing is not applicable to this article as no new data were generated or analyzed in this case report.
