# Supplementary material for: Digital Surgical Management of Traumatic Occlusal Disorder: A Case Report
Source: Case Rep Dent. 2026 Oct 21;2026:9103011. doi: 10.1155/crid/9103011 (PMC13685501; doi:10.1155/crid/9103011)
Supplement: Supplementary file 1 [file CRID-2026-9103011-s001.pdf]

CARE Checklist of information to include when writing a case report

CARE case report guidelines

(cc) BY-NC-ND

Topic	Item	Checklist item description	Reported on Line
Title	1	The diagnosis or intervention of primary focus followed by the words "case report"	page1
Key Words	2	2 to 5 key words that identify diagnoses or interventions in this case report, including "case report"	page2
Abstract (no references)	3a	Introduction: What is unique about this case and what does it add to the scientific literature?	page1
	3b	Main symptoms and/or important clinical findings	page1
	3c	The main diagnoses, therapeutic interventions, and outcomes	page1-2
	3d	Conclusion—What is the main "take-away" lesson(s) from this case?	page2
Introduction	4	One or two paragraphs summarizing why this case is unique (**may include references**)	page2-3
Patient Information	5a	De-identified patient specific information	page4
	5b	Primary concerns and symptoms of the patient	page4
	5c	Medical, family, and psycho-social history including relevant genetic information	page4
	5d	Relevant past interventions with outcomes	Not reported
Clinical Findings	6	Describe significant physical examination (PE) and important clinical findings	page4
Timeline	7	Historical and current information from this episode of care organized as a timeline	page4
Diagnostic Assessment	8a	Diagnostic testing (such as PE, laboratory testing, imaging, surveys)	page4
	8b	Diagnostic challenges (such as access to testing, financial, or cultural)	Not reported
	8c	Diagnosis (including other diagnoses considered)	page4
	8d	Prognosis (such as staging in oncology) where applicable	Not applicable
Therapeutic Intervention	9a	Types of therapeutic intervention (such as pharmacologic, surgical, preventive, self-care)	page4-5
	9b	Administration of therapeutic intervention (such as dosage, strength, duration)	page5
	9c	Changes in therapeutic intervention (with rationale)	Not reported
Follow-up and Outcomes	10a	Clinician and patient-assessed outcomes (if available)	page5
	10b	Important follow-up diagnostic and other test results	page5
	10c	Intervention adherence and tolerability (How was this assessed?)	Not reported
	10d	Adverse and unanticipated events	Not reported
Discussion	11a	A scientific discussion of the strengths AND limitations associated with this case report	page6-7
	11b	Discussion of the relevant medical literature **with references**	page6-7
	11c	The scientific rationale for any conclusions (including assessment of possible causes)	page7-8
	11d	The primary "take-away" lessons of this case report (without references) in a one paragraph conclusion	page8
Patient Perspective	12	The patient should share their perspective in one to two paragraphs on the treatment(s) they received	page5
Informed Consent	13	Did the patient give informed consent? Please provide if requested	Yes ✓ No
